# Arthritis is associated with high nutritional risk among older Canadian adults from the Canadian Longitudinal Study on Aging

**DOI:** 10.1038/s41598-024-58370-7

**Published:** 2024-05-11

**Authors:** Roxanne Bennett, Thea A. Demmers, Hugues Plourde, Kim Arrey, Beth Armour, Guylaine Ferland, Lisa Kakinami

**Affiliations:** 1 School of Health, Concordia University, Montreal, QC Canada; 2 École de Santé Publique, Centre de Recherche en Santé Publique, Université de Montréal, Montreal, QC Canada; 3 School of Human Nutrition, McGill University, Montreal, QC Canada; 4 HUMA+, Westmount, QC Canada; 5 PEN- Practice-Based Evidence in Nutrition®, Dietitians of Canada, Toronto, Canada; 6 Département de Nutrition, Faculté de Médicine, Université de Montréal, Montreal, QC Canada; 7 Department of Mathematics and Statistics, Concordia University, 1455 de Maisonneuve West, Montreal, QC H3G 1M8 Canada

**Keywords:** Epidemiology, Risk factors, Rheumatology

## Abstract

This study assessed the association between arthritis, functional impairment, and nutritional risk (NR). Cross-sectional data were from the Canadian Longitudinal Study on Aging, a nationally representative sample of 45–85-year-old community-dwelling Canadians (n = 41,153). The abbreviated Seniors in the Community: Risk Evaluating for Eating and Nutrition II (SCREEN II-AB) Questionnaire determined NR scores (continuous), and high NR (score < 38); the Older American Resources and Services scale measured functional impairment. NR scores and status (low/high) were modelled using multiple linear and logistic regressions, respectively. Analyses adjusted for demographic characteristics, functional impairment, and health (body mass index, self-rated general and mental health). Additional analyses stratified the models by functional impairment. People with arthritis had poorer NR scores (B: − 0.35, CI − 0.48, − 0.22; *p* < 0.05) and increased risks of high NR (OR 1.11, 95% CI 1.06, 1.17). Among those with functional impairment, the likelihood of high NR was 31% higher in people with arthritis compared to those without arthritis (95% CI 1.12, 1.53). Among those with no functional impairment, the likelihood of high NR was 10% higher in people with arthritis compared to those without (95% CI 1.04, 1.16). These relationships differed based on the type of arthritis. Arthritis is associated with high NR in community-dwelling older adults, both with and without functional impairment. Findings highlight the need for further research on these relationships to inform interventions and improve clinical practices.

## Introduction

In 2018, 46.9% of Canadians over the age of 65 years suffered from arthritis^1^. Recent evidence suggests that older adults affected by disabling conditions (such as arthritis) are more likely to suffer from malnutrition^2,3^. The relationship between nutritional status and arthritis is complex and may vary based on the type and severity of arthritis, as well as the affected joints^4–6^. For example, joint pain may impact dexterity or the ability to stand to prepare food, while the fatigue present in certain forms of arthritis may decrease the energy to cook or to eat^5–7^. Because malnutrition has been linked to increased morbidity and mortality in this age group, the early detection of vulnerable individuals may allow for timely interventions and improved outcomes^2,3,8–10^.

To help prevent progression to full-fledged malnutrition, screening tools have been designed to detect the presence of risk factors associated with poor nutritional status, also known as “nutritional risk”^8,11,12^. While the relationship between arthritis and functional impairment is established^13^, the contribution of different types of arthritis, such as osteoarthritis (OA), and other external factors to the development of functional impairment^14^, and further to the development of nutritional risk, is less understood. This is in part due to delays in diagnosis of arthritis^15,16^, which could be precipitated sooner by functional impairment in some cases, depending on the joint and personal habits, but not in others. It is also partly due to bidirectionality between nutritional risk and subsequent development of functional impairment. Both points contribute to difficulty in establishing the temporality needed to clarify the role of functional impairment as a mediator rather than a moderator within the relationship between arthritis and nutritional risk. Past research suggests that nutritional risk may be linked to functional impairment^2,8,17^. Specifically, limitations with certain activities of daily living (ADLs) and instrumental activities of daily living (IADLs), such as meal preparation, may be particularly impactful in a person’s susceptibility to nutritional risk^4,17–20^. Although an estimated 10%-59% of patients with arthritis experience difficulties during meal preparation, there is a limited body of research on the relationship between meal preparation and arthritis^4,19,21–24^. Previous research has linked impairment with certain ADLs and IADLs to both the number of painful joints and the severity of pain in individuals with arthritis^4,24^. These results are complemented by a 2016 study, which found an association between chronic musculoskeletal pain and nutritional risk in seniors^25^. While pain caused by arthritis is hypothesized to play an important role in arthritis-related functional impairment, its association with nutritional risk remains understudied^5,26,27^.

Thus, there is a paucity of data on the relationship between arthritis, nutrition risk, and the role of functional impairment within that relationship. This study aims to bridge this gap by (1) describing the association between arthritis and nutritional risk, (2) describing the association between arthritis and nutritional risk while considering meal preparation impairment, and (3) assessing the relationship between functional impairment and these associations. To address these objectives, a large representative sample of older Canadian adults was used.

## Methods

### Data source

The Canadian Longitudinal Study on Aging (CLSA) is a nationally representative cohort study. Study design and measures have been published but are briefly described here^28^. Baseline data (2010–2015) were from over 50,000 Canadians between the ages of 45 and 85 years from all provinces (excluding territories). Individuals who were institutionalized, incarcerated, lived on a reserve, or had cognitive impairment at baseline were ineligible to participate. The study was conducted according to the guidelines laid down in the Declaration of Helsinki and all procedures involving research study participants were approved by the CIHR Advisory Committee on Ethical, Legal and Social Issues, and the research boards at the study sites. Ethics approval for this secondary data analysis was obtained from Concordia University (#30007632). Written informed consent was obtained from all participants. This analysis includes data from both the “tracking” group (self-reported data during a telephone interview, n = 21,241) and the “comprehensive” group (additionally includes onsite measurements, tests, questionnaires, and in-person home interviews, n = 30,097)^28^.

Participants were excluded from this secondary data analysis if they had missing data for total household income (n = 3321), number of people living in the household (n = 23), education (n = 2421), self-rated general health (n = 36), self-rated mental health (n = 33), body mass index (BMI) (n = 204), and any question within the nutritional risk or functional impairment questionnaires (n = 4147). In total, 41,153 respondents were included in this study.

### Measures

#### Arthritis

Respondents were asked if they had ever been told by a doctor that they had any of the following: OA of the knee(s), the hip(s), the hand(s), rheumatoid arthritis (RA), or any other form of arthritis. Respondents who did not know about a positive arthritis diagnosis (n = 569) were considered not to have arthritis. Thus, in both groups combined (“tracking” and “comprehensive”), 14,468 respondents were considered to have arthritis and 26,685 were considered not to have arthritis. Among those with arthritis, people were additionally categorized into three non-mutually exclusive groups with OA (n = 10,485), RA (n = 1510) and other forms of arthritis (n = 4901).

In addition to self-reported arthritis, the “comprehensive” group also included validated disease ascertainment algorithms that determined the likelihood of OA of the hands, hips, and knees based on self-reported symptoms and clinical observations^29^. Participants were categorized based on whether they responded affirmatively or negatively to experiencing symptoms such as joint enlargement and pain. As algorithms were only available for OA in the “comprehensive” group (n = 25,099), the relationships between OA-related pain and nutrition were assessed in sensitivity analyses.

#### Nutritional risk

Nutritional risk was measured using the Seniors in the Community Risk Evaluation for Eating and Nutrition II—Abbreviated (SCREEN II-AB, also known as SCREEN-8). The SCREEN II-AB is a validated 8-item tool designed for epidemiological and clinical use in community-dwelling seniors^11,30^. The measure attributes scores to: recent weight changes (range: 0–8), frequency of meal skipping (0–8), general appetite (0–8), difficulties with swallowing (0–8) daily vegetable and fruit consumption (0–4), daily fluid intake (0–4), the social context of mealtime (0–4), and the frequency of cooking meals at home (0–4)^30,31^, for a maximum score of 48. Lower SCREEN II-AB scores reflected higher nutritional risk^32^. High nutritional risk (H-NR) was defined as a score below 38 as this cut-off had optimal sensitivity and specificity in identifying nutritional risk when compared to a dietitian’s clinical assessment (including medical and nutritional history, dietary intake and anthropometry)^11^.

#### Impairment

Participants’ functional impairment was measured using the Older Americans’ Resources and Services (OARS) Multidimensional Functional Assessment Questionnaire^20^. This validated questionnaire includes items about seven ADLs (dressing, eating, appearance upkeep, walking, bathing, getting in and out of bed, using the bathroom) and seven IADLs (meal preparation, using the telephone, travelling, shopping, housework, taking medication, financial management)^20^. Response options included “required no help”, “some help”, or are “unable to perform” without assistance^20^. Because few CLSA participants reported any functional impairment, the total OARS score was then used to categorize the respondents’ overall functional capacity as dichotomous (no help required for any activity, as a referent, versus some help or unable to perform at least one activity without assistance), similarly to a previous study using these data^33^. To isolate its effect as both an independent predictor and a covariate of interest, meal preparation impairment was considered separately from other ADLs and IADLs in the OARS scale. Thus, functional impairment was subdivided into (1) impairment specific to meal preparation, and (2) impairment to any activity (excluding meal preparation).

#### Covariates

Covariates of interest were identified based on the findings of past research on arthritis, chronic disability, and food insecurity^34–36^. These covariates included demographic characteristics such as age, sex, race (white vs non-white), total household income, education, and the number of individuals residing in the household. Total annual household income was categorized as less than $20,000, $20,000–$49,999, $50,000–$100,000, and greater than $100,000 (CAD). Education was considered as: less than secondary school, secondary school, trade school, and university or higher. Health covariates included BMI, self-rated mental health, and self-rated general health. Given the documented protective effects of a higher BMI in adults over 65 years, BMI was categorized according to the Global Leadership Initiative on Malnutrition (GLIM) criteria for malnutrition, with underweight (< 20 kg/m^2^ for people aged < 70 and < 22 kg/m^2^ for those aged >= 70), normal to overweight as the referent (20–29.9 kg/m^2^ for people aged < 70 and 22–29.9 kg/m^2^ for those aged >= 70), or obese (≥ 30.0 kg/m^2^)^37^. BMI was calculated from self-reported height and weight in the “tracking” group and measured height and weight in the “comprehensive” group. Self-rated general health scores and self-rated mental health scores were each coded into two groups with the first including those who self-rated as “excellent” and “very good” and the latter including those who self-rated as “good”, “fair”, or “poor”.

### Statistical analysis

All analyses were conducted with SAS version 9.4 (SAS Institute Inc., Cary, NC, USA) and incorporated survey weights in accordance with the CLSA recommendations. Descriptive statistics compared the sample’s demographic and health characteristics to those who were excluded. Hierarchical multiple linear regression was used to model nutritional risk scores (where lower scores indicated greater risk) from the SCREEN-II-AB. Additionally, H-NR using the SCREEN II-AB cut-off score of 38 was assessed with logistic regression models^11,31^. Regressions were entered in three steps, with covariates as described previously. The first step (Model 1) controlled for demographic characteristics (age, sex, race, total household income, number of people in the household, and education) and measures of health (BMI, self-rated general physical and mental health). A second model (Model 2) additionally adjusted for meal preparation impairment. The third model (Model 3) further controlled for general functional impairment (excluding meal preparation).

Betas for arthritis (any vs none; as well as based on the three non-mutually exclusive groups as described previously), meal preparation impairment, and functional impairment from linear regression models assessing nutritional risk scores (continuous) and Odds Ratios for logistic regression models assessing probability of high nutritional risk (< 38 vs >=38) are all presented. These data are cross-sectional; as temporal precedence cannot be established, tests for mediation may be unwarranted and only *indirect* effects of *partial* mediation can be calculated^38^. In accordance to the methodological guidelines in the literature, the difference in coefficients between Model 3 and Model 1 and its standard error was calculated. The estimate was compared to the t-distribution (df = 41,118), with the null hypothesis being that the effect was mediated through functional impairment^39–41^. Lastly, based on evidence of an interaction between arthritis and functional impairment, the regression models were also stratified by functional impairment for further comparison. All analytical procedures were repeated for the sensitivity analysis investigating OA-related pain as previously described.

### Ethical approval

The study was conducted according to the guidelines laid down in the Declaration of Helsinki and all procedures involving research study participants were approved by the CIHR Advisory Committee on Ethical, Legal and Social Issues, and the research boards at the study sites. Ethics approval for this secondary data analysis was obtained from Concordia University (#30007632).

### Informed consent

Written informed consent was obtained from all participants.

## Results

Compared to the participants who were excluded from the sample, those included in the analysis were more likely to be women (49.8% vs 44.3%, *p* < 0.0001), younger (59.2 years vs 63.2; *p* < 0.0001), less likely to have arthritis (32% vs 39%, *p* < 0.0001), and had higher (better) nutritional scores (39.2 vs 37.6; n = 46,410; *p* < 0.0001; data not shown).

Demographic characteristics of participants with and without arthritis differed for all measured characteristics (Table 1); the sample with arthritis was older (62.4 vs 57.8 years, *p* < 0.0001) and had a lower proportion of males (41.6% vs 53.7%, *p* < 0.0001). Both groups differed significantly on educational attainment and income. Respondents with arthritis had greater nutritional risk than those without arthritis, indicated by their lower nutritional risk scores (38.5 vs 39.6, *p* < 0.0001) and greater proportion at high nutritional risk (37.9% vs 31.2%, *p* < 0.0001). Moreover, participants with arthritis had greater levels of general functional impairment (13.1% vs 4.8%, *p* < 0.0001) and meal preparation impairment (0.7% vs 0.3%, *p* < 0.0001). People with arthritis had higher proportions of obesity compared to those without arthritis (*p* < 0.0001).

**Table 1:** Sociodemographic and health characteristics of participants with and without self-reported arthritis from the Canadian Longitudinal Study on Aging baseline survey (n = 41,153).

	Arthritis^a^ (n = 14,468)	No arthritis (n = 26,685)	*p*
Sociodemographic characteristics			
Male, %	41.6	53.7	< 0.0001
Age in years, Median (SD)	62.4 (0.1)	57.8 (0.06)	< 0.0001
White^b^, %	96.6	95.7	< 0.0001
Income, %			
< 20 K/year	4.9	3.0	< 0.0001
20–50 K/year	24.3	16.8	
50–100 K/year	37.3	34.4	
100 K+/year	33.6	45.8	
Highest education, %			
Less than secondary	5.2	3.6	< 0.0001
Secondary	20.0	17.0	
Trade school	34.7	32.7	
University or higher	40.1	46.6	
Num. of people living in the household, Median (SD)	1.6 (0.002)	1.8 (0.002)	< 0.0001
Health characteristics			
Nutritional score	38.5 (0.08)	39.6 (0.05)	< 0.0001
High nutritional risk, %	37.9	31.2	< 0.0001
Any meal preparation impairment, %	0.7	0.3	< 0.0001
Any functional impairment (excl. meal preparation), %	13.1	4.8	< 0.0001
Number of functional impairments (excl. meal preparation), % (n = 3,599)			
0	87	95.3	< 0.0001
1	12.0	4.4	
2	0.7	0.2	
3	0.2	0.07	
4	0.1	0.03	
Excellent or very good physical health, %	53.0	67.4	< 0.0001
Excellent or very good mental health, %	66.6	73.2	< 0.0001
Weight status, %			
Underweight	3.8	3.8	< 0.0001
Normal-weight or overweight	61.9	72.2	
Obese	34.3	23.9	

Weighted frequencies and means. Percentages may not add up to 100 due to rounding.

^a^Types of arthritis: osteoarthritis (OA) of the hand (n = 5261), OA of the hip (n = 3426), OA of the knee (n = 6019), rheumatoid arthritis (n = 1510), other form of arthritis (n = 4901). Sum of these types exceeds total number of individuals with arthritis due to the possibility of reporting more than one type of arthritis.

^b^n = 41,120.

From the multiple linear and logistic regression models, those living with arthritis were associated with a worse nutritional risk score (Table 2; Model 1; B = − 0.43, CI − 0.57, − 0.30; *p* < 0.05) and H-NR (Model 1; OR 1.14 CI 1.08, 1.20; *p* < 0.05) after controlling for demographic and health characteristics. Both associations from the first model remained significant after further adjustment. Respondents with arthritis had nutritional risk scores that were worse (B = − 0.35, CI − 0.48, − 0.22; *p* < 0.05) and were 11% more likely to have H-NR (CI 1.06, 1.17; *p* < 0.05) after controlling for both meal preparation impairment and functional impairment (Model 3). Nutritional risk scores were worse among those with RA (B = − 0.99, CI − 1.36, − 0.62; *p* < 0.05). Functional impairment in individuals regardless of arthritis was associated with a 1.88-point decrease in nutritional risk score (Model 3; CI − 2.16, − 1.62; *p* < 0.05) and a 61% higher likelihood of H-NR (Model 3; CI 1.48, 1.75; *p* < 0.05).

**Table 2:** Multivariable linear and logistic regressions assessing nutritional risk based on arthritis, meal preparation impairment (MPI) and functional impairment (FI, excluding meal preparation impairment) of respondents from the Canadian Longitudinal Study on Aging baseline survey (n = 41,120).

	Model 1		Model 2		Model 3	
	B^a,d^ (95% CI)	OR^a,e^ (95% CI)	B^b,d^ (95% CI)	OR^b,e^ (95% CI)	B^c,d^ (95% CI)	OR^c,e^ (95% CI)
Arthritis defined as binary (yes/no)						
Arthritis^f^ versus no arthritis	**− 0.43 (− 0.57, − 0.30)**	**1.14 (1.08, 1.20)**	**− 0.43 (− 0.56, − 0.29)**	**1.14 (1.08, 1.20)**	**− 0.35 (− 0.48, − 0.22)**	**1.11 (1.06, 1.17)**
MPI versus no MPI			**− 2.66 (− 3.65, − 1.68)**	**1.85 (1.35, 2.54)**	**− 1.34 (− 2.33, − 0.34)**	1.33 (0.96, 1.83)
FI versus no FI					**− 1.88 (− 2.16, − 1.62)**	**1.61 (1.48, 1.75)**
Arthritis defined as non-mutually exclusive groups (RA/OA/Other/None)						
RA versus none	**− 1.09 (− 1.46, − 0.71)**	**1.34 (1.19, 1.51)**	**− 1.08 (− 1.45, − 0.70)**	**1.33 (1.18, 1.50)**	**− 0.99 (− 1.36, − 0.62)**	**1.31 (1.16, 1.48)**
OA versus none	**− 0.26 (− 0.41, − 0.12)**	**1.10 (1.0.4, 1.16)**	**− 0.26 (− 0.41, − 0.11)**	**1.10 (1.04, 1.16)**	**− 0.18 (− 0.33, − 0.03)**	**1.08 (1.02, 1.14)**
Other versus none	**− 0.45 (− 0.65, − 0.25)**	**1.11 (1.03, 1.19)**	**− 0.45 (− 0.64, − 0.25)**	**1.11 (1.03, 1.19)**	**− 0.39 (− 0.59, − 0.19)**	**1.09 (1.01, 1.17)**
MPI versus no MPI			**− 2.65 (− 3.64, − 1.66)**	**1.84 (1.34, 2.53)**	**− 1.34 (− 2.34, − 0.35)**	**1.33 (0.96, 1.84)**
FI versus no FI					**− 1.87 (− 2.14, − 1.60)**	**1.60 (1.45, 1.74)**

^a^Model 1: Adjusted for age, sex, education, household income, race, BMI category, self-rated general health, and self-rated mental health.

^b^Model 2: Model 1 with further adjustment for meal preparation impairment.

^c^Model 3: Model 2 with further adjustment for general functional impairment.

^d^Betas and standard errors from multiple linear regression models.

^e^Odds ratios and 95% confidence intervals from multiple logistic regression models.

^f^Types of arthritis: osteoarthritis (OA) (n = 10,485), rheumatoid arthritis (n = 1510), other form of arthritis (n = 4901). Sum of these types exceeds total number of individuals with arthritis due to the possibility of reporting more than one type of arthritis.

Bold: statistical significance at *p* < 0.05.

Stratification by functional impairment indicated that the association between arthritis and nutritional risk score differed based on whether the individual had functional impairment or not (Table 3). For instance, while arthritis was associated with a nutritional risk score that was 0.30 units lower among people with no functional impairment (Model 1; CI − 0.44, − 0.16; *p* < 0.05), the relationship was more severe among those with functional impairment (Model 1; B = − 0.90, CI − 1.41, − 0.38; *p* < 0.05). Compared to individuals without arthritis, people with arthritis had increased odds of H-NR whether they experienced functional impairment (Model 2; OR 1.31, CI 1.12, 1.53; *p* < 0.05) or not (Model 2; OR 1.10, CI 1.04, 1.16; *p* < 0.05). Among those with arthritis but no functional impairment, those with RA had the highest increased odds of H-NR (Model 2; OR 1.29, CI 1.13, 1.47; *p* < 0.05), H-NR risks were exacerbated among those who experienced functional impairment (Model 2; OR 1.41, CI 1.06, 1.87; *p* < 0.05). The indirect effect of functional impairment on nutritional risk was estimated at − 0.08 (SE: 0.10; *p* > 0.05).

**Table 3:** Multivariable linear and logistic regression on nutritional risk stratified by functional impairment (FI) based on arthritis of respondents from the Canadian Longitudinal Study on Aging baseline survey (n = 41,120).

	Model 1		Model 2	
	B^a,c^ (95% CI)	OR^a,d^ (95% CI)	B^b,c^ (95% CI)	OR^b,d^ (95% CI)
Stratified by FI				
With FI (n = 3597): Arthritis^e^ versus no Arthritis	**− 0.90 (− 1.41, − 0.38)**	**1.31 (1.11, 1.53)**	**− 0.90 (− 1.42, − 0.38)**	**1.31 (1.12, 1.53)**
No FI (n = 37,523): Arthritis versus no Arthritis	**− 0.30 (− 0.44, − 0.16)**	**1.10 (1.04, 1.16)**	**− 0.30 (− 0.44, − 0.16)**	**1.10 (1.04, 1.16)**
With FI (n = 3597)				
RA versus none	**− 1.46 (− 2.48, − 0.45)**	**1.41 (1.06, 1.86)**	**− 1.46 (− 2.48, − 0.45)**	**1.41 (1.06, 1.87)**
OA versus none	**− 0.55 (− 1.07, − 0.03)**	**1.27 (1.08, 1.49)**	**− 0.56 (− 1.07, − 0.04)**	**1.27 (1.09, 1.49)**
Other versus none	− 0.66 (− 1.32, 0.007)	1.12 (0.92, 1.36)	− 0.66 (− 1.33, 0.002)	1.12 (0.92, 1.37)
No FI (n = 37,523)				
RA versus none	**− 0.88 (− 1.27, − 0.48)**	**1.29 (1.13, 1.47)**	**− 0.88 (− 1.27, − 0.48)**	**1.29 (1.13, 1.47)**
OA versus none	− 0.14 (− 0.29, 0.01)	1.06 (0.99, 1.13)	− 0.14 (− 0.29, 0.01)	1.06 (1.00, 1.13)
Other versus none	**− 0.35 (− 0.55, − 0.15)**	**1.09 (1.01, 1.17)**	**− 0.35 (− 0.55, − 0.15)**	**1.09 (1.01, 1.17)**

^a^Model 1: Adjusting for age, gender, education, household income, race, BMI category, self-rated general health, and self-rated mental health.

^b^Model 2: Model 1 with further adjustment for meal preparation impairment (MPI).

^c^Betas and standard errors from multiple linear regression models.

^d^Odds ratios and 95% confidence intervals from multiple logistic regression models.

^e^Types of arthritis: osteoarthritis (OA) (n = 10,485), rheumatoid arthritis (n = 1510), other form of arthritis (n = 4901). Sum of these types exceeds total number of individuals with arthritis due to the possibility of reporting more than one type of arthritis.

Bold: statistical significance at *p* < 0.05.

### Sensitivity analysis: OA-related pain and nutritional risk

The “comprehensive” group was significantly younger than the “tracking” group (58.9 vs 59.7 years, *p* < 0.0001), had a higher proportion of males (50.3% vs 49.5%, *p* = 0.03), a lower proportion of people with arthritis (30.0% vs 34.9%, *p* < 0.0001), and lower proportion of people with functional impairment (6.7% vs 8.3%, *p* < 0.0001). The groups did not differ in meal-related impairment nor in the likelihood of H-NR. Results from the pain sensitivity analysis conducted among those from the “comprehensive” group were consistent in both direction and magnitude with that of the main analyses (data not shown).

## Discussion

Arthritis has previously been linked to nutritional problems, such as poor diet quality, malnutrition, and food insecurity^19,23^. This increased risk of nutritional problems may stem from the complex interplay between physiological/physical, psychological, and social factors associated with arthritis^5^. For example, systemic inflammation resulting from inflammatory arthritis (which includes diseases such as RA) has been shown to trigger muscle loss and has been associated with greater pain and fatigue, thus potentially triggering physical and psychological barriers to adopting a healthy diet^5,42^. Osteoarthritis, traditionally perceived as a “wear and tear” condition, can cause significant pain with certain tasks and a loss of dexterity, with increasing evidence also pointing to the presence of a chronic low-grade inflammation^43–45^. Behavioral and psychological contributors to nutritional problems in people with arthritis include a higher incidence of depression, often associated with pain and fatigue, which may impact food intake and diet quality, while external factors include reduced accessibility to food and reliance on social support to cook or grocery shop^2,5,46–48^.

However, the generalizability of the past research has often been limited by small sample sizes and emphasis on RA^8,19,22,23,49,50^. In this large, representative sample of older Canadian adults, having any arthritis was associated with poorer nutritional risk scores and an increased likelihood of being at high nutritional risk and the association was strongest among those with RA. This association was maintained after adjustment for meal preparation impairment and general functional impairment. Functional impairment partially mediated the relationship between arthritis and nutritional risk. While having arthritis was associated with nutritional risk, the nutritional risk scores were poorer among those who also had functional impairment. Stratification for functional impairment revealed that respondents with arthritis were more likely to present lower SCREEN II-AB scores and were more likely to be at high nutritional risk, even in the absence of any functional impairment.

This is the first study to investigate the association between arthritis and nutritional risk specifically; however, past research may provide important context for its findings. In 2013, a study using data from the Canadian Community Health Survey (CCHS), the precursor to the CLSA, found that disability was independently linked with increased nutritional risk in Canadian seniors^8^. The CCHS results were echoed by research conducted outside of Canada which reinforces the positive association between nutritional risk and disability in older adults^51–53^. However, the absence of referents without arthritis in these previous studies has limited the interpretation of results for those with arthritis but no functional impairment^19,21,22^. We addressed this gap with a large representative sample encompassing those with and without arthritis, as well as those with and without functional impairment.

Variability in arthritis-related symptoms may also influence the type and severity of limitations with specific ADLs and IADLs^2,4,24^. Previous studies of older adults have shown that difficulties with meal preparation and shopping for food were the ADLs and IADLs most highly correlated with nutritional risk^17,54^. In our study, the pain sensitivity analysis utilizing a thorough assessment based on joint-related pain was consistent with the main findings. Nevertheless, affected joints may differentially contribute to functional impairment and the subsequent meal preparation adaptations should be further explored^2^.

Our results contribute to the literature to guide the development of interventions for people living with arthritis, thus it is of practical importance to consider the concepts of moderators, mediators and their role in mechanisms of producing an outcome. A moderator may be a mediator and vice-versa, what distinguishes the two is the inference of causality; strong evidence, temporality and a lack of measurement error are needed to label a predictor as a mediator^38,39^. The literature suggests that functional impairment may be both a moderator, and a mediator for the relationship between arthritis and nutritional risk^39^. Indeed, in this study, we found evidence of both. It is theorized that having arthritis restricts functional abilities necessary for maintaining an adequate diet^4,19,22^. As the cross-sectional nature of this study prevents any inference of causality, we were only able to calculate whether functional impairment could be a partial mediator^39,40^. In particular, mounting evidence suggests that the relationship between nutritional risk and disability is bidirectional, as nutritional risk (i.e. skipping meals, weight change, appetite, swallowing and eating habits) may, in turn, aggravate certain symptoms (i.e. fatigue, physical activity) that contribute to functional decline^2,8,9^. As temporality is a necessary condition for mediation analyses to be conducted properly^38^, future longitudinal research investigating potential mediation of functional impairment of nutrition risk for people with arthritis is needed^13^.

This study has certain limitations that may reduce the applicability of its results. Firstly, all data were from the baseline wave of the CLSA; follow-up is ongoing but was not available at the time of this project. Similarly, the analytic sample was significantly different from the participants who were excluded from the analysis in demographic and health characteristics and results cannot be generalized to the entire CLSA sample. As the excluded participants were more likely to have arthritis and lower (poorer) nutritional scores, suggesting that our results may underestimate their relationships. Additionally, the use of self-reported data might be affected by both recall and social desirability bias. Although this study adjusted for age in all regression models, those with arthritis were approximately four years older than those without arthritis. As older adults’ increased susceptibility to poor nutritional status stems from numerous complex physiological changes such as altered metabolism, cognitive decline, and changing socioeconomic factors^2,3,8–10^, a better understanding of the interactions between aging and functional impairment on diet is needed.

While this study had a large, representative sample, only a small number of respondents experienced meal-related ADL impairment, which may have rendered any effects on nutritional risk statistically undetectable. Similarly, of those who reported having difficulties with other ADLs, the majority reported only one difficulty. This limited our ability to assess a potential linear relationship with number of ADL difficulties as a continuous score. As the participants included in this analysis were younger, less likely to have arthritis, and had better nutritional risk scores than those who were excluded, the results reported here are likely underestimates.

Although the CLSA data collected information on type and severity of arthritis, these details were assessed differently between the tracking (self-reported) and comprehensive (based on disease ascertainment algorithms) groups. Our sensitivity analysis on whether pain OA-related pain and nutrition were consistent with our main findings. Nevertheless, whether the severity of arthritis, and the affected joints impacts nutritional risk should be further explored in a future study. We assessed whether the arthritis type (RA, OA, or Other) were differentially associated with nutritional risk and found that those with RA were likely the most severely impacted. However, these were non-mutually exclusive groups, as participants could have more than one type of arthritis. Considering the numerous types of arthritis and the variability in their symptoms, further research on differentiation between conditions is needed.

The SCREEN II-AB is a practical, accessible screening tool for the detection of a broad range of nutritional issues^8,11,49^. Previous research using the SCREEN II in older adults found that it was more inclusive than other screening tools, such as the SNAQ^65+^, and included determinants of early malnutrition that included both over- and undernutrition^55^. In a study using the 2008/2009 CCHS, nutritional risk was associated with disability, medication use, living alone and low social support in adults aged 65 years^8^. In a New Zealand study of 655 adults, being at high nutritional risk was linked to lower physical health scores, higher depression scores, more difficulty accessing shops, and higher likelihood of living on only a limited pension^56^. Despite its high specificity and sensitivity, the SCREEN II-AB provides neither a diagnosis nor a measure of diet quality. As there is no gold standard for measuring nutritional risk^8,49^, further work on identifying which nutrition-related behaviors are most problematic for respondents with and without arthritis is needed.

## Conclusion

The results of this study highlight the presence of an association between arthritis with poorer nutritional risk scores and higher nutritional risk in a nationally representative sample of Canadian adults between the ages of 45 and 85 years. There is increasing evidence indicating that the relationship between nutrition and arthritis is multifactorial, likely caused by complex interactions between physical, psychological, social, financial, and environmental factors^35,47,50,57,58^. The overlap of these characteristics warrants an intersectional perspective, especially as there is evidence separately linking each of these factors to functional status, disease activity, and dietary behaviors^35,50,57,59^. More research is necessary to understand the relationship between arthritis and nutrition in specific groups to inform adapted preventative interventions and improve clinical practices.

## Data Availability

The data that support the findings of this study are available from the Canadian Longitudinal Study on Aging (CLSA) but restrictions apply to the availability of these data, which were used under license for the current study, and so are not publicly available. Data are however available from the CLSA, or from the authors upon reasonable request and with permission of the CLSA.
